# Antibody Epitopes of Pneumovirus Fusion Proteins

**DOI:** 10.3389/fimmu.2019.02778

**Published:** 2019-11-29

**Authors:** Jiachen Huang, Darren Diaz, Jarrod J. Mousa

**Affiliations:** ^1^Department of Infectious Diseases, College of Veterinary Medicine, University of Georgia, Athens, GA, United States; ^2^Center for Vaccines and Immunology, College of Veterinary Medicine, University of Georgia, Athens, GA, United States

**Keywords:** RSV, respiratory syncytial virus, human metapneumovirus, hMPV, antibody—antigen complex, X-ray crystallography, pneumovirus infections

## Abstract

The pneumoviruses respiratory syncytial virus (RSV) and human metapneumovirus (hMPV) are two widespread human pathogens that can cause severe disease in the young, the elderly, and the immunocompromised. Despite the discovery of RSV over 60 years ago, and hMPV nearly 20 years ago, there are no approved vaccines for either virus. Antibody-mediated immunity is critical for protection from RSV and hMPV, and, until recently, knowledge of the antibody epitopes on the surface glycoproteins of RSV and hMPV was very limited. However, recent breakthroughs in the recombinant expression and stabilization of pneumovirus fusion proteins have facilitated in-depth characterization of antibody responses and structural epitopes, and have provided an enormous diversity of new monoclonal antibody candidates for therapeutic development. These new data have primarily focused on the RSV F protein, and have led to a wealth of new vaccine candidates in preclinical and clinical trials. In contrast, the major structural antibody epitopes remain unclear for the hMPV F protein. Overall, this review will cover recent advances in characterizing the antigenic sites on the RSV and hMPV F proteins.

## Introduction

The recently reclassified *Pneumoviridae* virus family includes the human pathogens respiratory syncytial virus (RSV) and human metapneumovirus (hMPV) (1). These viruses are among the most common causes of childhood respiratory tract infection (2). Severe disease primarily occurs in young children, the elderly, and the immunocompromised, and reinfection can occur throughout childhood and adulthood, as sterilizing immunity is not acquired after infection. Both viruses exhibit genetic stability, with relatively few changes in viral sequences among circulating strains. Despite decades of research, there are no approved vaccines to prevent pneumovirus infection. Fortunately, a wave of new progress in recent years has led to the development of new vaccine candidates and therapeutics, largely due to breakthroughs in structural biology and immunological techniques. This review will cover recent findings on antigenic epitopes of RSV and hMPV fusion glycoproteins.

## Global Burden of Pneumoviruses

### Respiratory Syncytial Virus

RSV is an enveloped, negative-sense, single stranded RNA virus, first isolated in 1955 from chimpanzees with respiratory illness (3), and subsequently isolated from infants with lower respiratory tract infection (4, 5). RSV is the leading cause of viral bronchiolitis and viral pneumonia in infants and children (6, 7), and nearly all children have been exposed to RSV before the age of 2 (8). RSV infection causes flu-like symptoms, bronchiolitis, and pneumonia that can be fatal to children. In addition, RSV infection poses a substantial threat to elderly populations and immunocompromised adults (9). RSV is highly contagious, and can be transmitted through direct contact or aerosol (10). Although numerous vaccines have undergone clinical trials (11), the monoclonal antibody (mAb) palivizumab remains the only approved therapeutic for RSV infection. Palivizumab has shown moderate efficacy at preventing RSV hospitalizations and intensive care unit admissions (12), however, the drug is only approved for prophylactic use, and in limited cases.

### Human Metapneumovirus

hMPV was identified in 2001 in the Netherlands from samples collected from 28 children with respiratory tract infection (13). The clinical features of hMPV infection are virtually identical to RSV, and display as mid-to-upper respiratory tract infection, and can be severe enough to cause life-threatening bronchiolitis and pneumonia. Infants and the elderly are the major groups for which hMPV infection may require hospitalization (14–18). In addition, hMPV infection can be severe in immunocompromised patients such as lung transplant (19) and hematopoietic stem-cell transplant recipients (20–23), and can cause febrile respiratory illness in HIV-infected patients (24) as well as exacerbate chronic obstructive pulmonary disease (25). Nearly 100% of children are seropositive by 5 years of age. There are currently no vaccines to prevent hMPV infection, and unlike the related pathogen respiratory syncytial virus (RSV), for which the prophylactic treatment palivizumab (26) is available for high-risk infants, no treatment or prophylaxis is available for hMPV.

## The Pneumovirus Fusion Protein

Pneumoviruses have three surface glycoproteins: the (F) fusion, (G) attachment, and small hydrophobic (SH) proteins, and the pneumovirus F protein is absolutely critical for viral infectivity. Antibodies are highly important for pneumovirus immunity (27, 28), and both RSV F and RSV G elicit neutralizing antibodies (29), while only antibodies to hMPV F are neutralizing (30). The pneumovirus F proteins belong to the family of class I viral fusion proteins that mediate the fusion of viral envelope and cell membrane during infection (31). The RSV F protein is first expressed as a F_0_ precursor, which is then cleaved at two furin cleavage sites in the trans-Golgi network to become fusion competent, generating the N-terminal F_2_ subunit and the C-terminal F_1_ subunit, while the p27 fragment in between F_1_ and F_2_ is removed. In contrast, hMPV F is cleaved at one site by different intracellular enzymes than RSV (32). Cleaved pneumovirus F proteins are anchored on the viral envelope by the trans-membrane domain of F_1_. The F_1_ and F_2_ fragments are covalently linked via two disulfide bonds, and the proteins form a trimeric structure consisting of three of the disulfide-linked fragments. The Pneumovirus F proteins fold into a pre-fusion conformation that contains a buried fusion peptide. Upon activation, the F protein undergoes a series of conformational changes leading to the post-fusion conformation in concert with cell-virus membrane fusion (31). The pre-fusion conformation of the pneumovirus F protein is unstable, and refolding can occur spontaneously or under certain stimuli that irreversibly transform the globular pre-fusion F into the elongated post-fusion formation. During the process of the pre-to-post-fusion conformational change, the highly hydrophobic fusion peptide located at the N terminus of F2 will insert into host cell membrane, forming a hairpin structure that bridges the two membranes together before a refolding event causes membrane fusion.

Until recently, knowledge on the structural aspects of pneumovirus fusion proteins was severely lacking, primarily due to instability of the pre-fusion conformation when recombinantly expressed. An X-ray crystal structure of the post-fusion conformation of RSV F was determined in 2011 by removal of the fusion peptide in the construct used for crystallization (33, 34). A breakthrough in 2013 facilitated structural-determination of the RSV F protein in the pre-fusion conformation by co-expression of RSV F with the mAb Fab fragment D25 to trap the protein in the pre-fusion state (35). This subsequently led to stabilization of the RSV F protein in the pre-fusion conformation by locking the protein in the pre-fusion state via artificial disulfide-bond insertion in addition to cavity-filling mutations (the Ds-Cav1 construct) (36). Following this, an additional pre-fusion-stabilized protein was generated in an alternative approach using the substitution of proline residues in the refolding regions and expression of the protein as a single-chain through the introduction of a glycine-serine linker (the SC-TM construct) (37). For hMPV, a partial X-ray crystal structure of hMPV F in the pre-fusion conformation in complex with the neutralizing Fab DS7 was determined in 2012 (38). Following the success with stabilization of pre-fusion RSV F, crystal structures of trimeric hMPV pre-fusion and post-fusion hMPV F were determined (39, 40). Pre-fusion hMPV F was stabilized with proline-substitutions to prevent refolding to the post-fusion conformation, while post-fusion hMPV F required the addition of a trimerization domain. Both hMPV F constructs required cleavage-site modification and co-expression with furin in CV-1 cells to generate fully-cleaved trimeric proteins. In addition to the structures described above, similar strategies were utilized to stabilize the parainfluenza virus fusion proteins, and bovine RSV F in the pre-fusion state (41, 42).

## Antigenic Differences Between RSV and hMPV F

**Figure 1:**
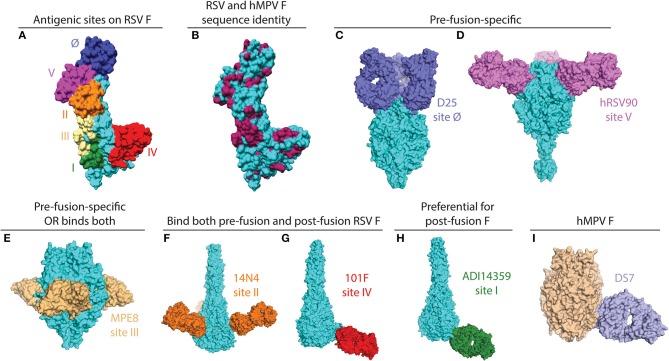
X-ray crystal structures of pneumovirus F proteins alone and in complex monoclonal antibody fragments. **(A)** The main antigenic sites on the RSV F protein are depicted on a monomer of the F protein (PDB ID: 5C6B). **(B)** The same structure as in **(A)** is colored according to sequence conservation with hMPV F. Conserved residues are shown in maroon, and primarily reside within antigenic sites III, IV, and V. Sequences of RSV and hMPV F were derived from PDB IDs: 5C6B and 5WB0. **(C)** The pre-fusion specific antibody D25 binds at the apex of pre-fusion RSV F (PDB ID: 4JHW). **(D)** hRSV90 binds antigenic site V and is pre-fusion-specific (PDB ID: 5TPN). **(E)** MPE8 is pre-fusion-specific and cross-reactive with hMPV F (PDB ID: 5U68). **(F)** 14N4 is a human antibody that targets antigenic site II and binds both pre-fusion and post-fusion RSV F (PDB ID: 5J3D). **(G)** The humanized mouse mAb, 101F, binds at antigenic site IV. For this structure, the crystal structure of 101F binding the site IV peptide (PDB ID: 3O41) was aligned with antigenic site IV of the post-fusion RSV F protein (3RRR). **(H)** The non-neutralizing site I mAb ADI14359 was isolated from an RSV-infected infant, and is preferential for post-fusion RSV F (PDB ID: 6APB). **(I)** DS7 is a hMPV F-specific mAb that was co-crystallized with a fragment of pre-fusion hMPV F. The structure of the DS7-F complex (PDB ID: 4DAG) is overlaid onto the trimeric pre-fusion hMPV F structure (PDB ID: 5WB0). All antibodies are colored in accordance with the text label, the RSV F protein is shown in cyan, and the hMPV F protein is shown in wheat. All figures and alignments were prepared in PyMol and Chimera.

The RSV and hMPV F proteins share ~30% sequence identify, and among the antigenic sites on RSV F, at least two are shared with hMPV F (antigenic sites III and IV) as a result of this conservation (Figures 1A,B). Despite the shared sequence conservation, several distinct features influence the differing antibody response to these viruses. The majority of RSV neutralizing activity in human sera is mediated by pre-fusion-specific RSV F antibodies (43), while the majority of hMPV neutralizing activity is mediated by antibodies recognizing both pre-fusion and post-fusion conformations (40). In addition, vaccination with pre-fusion RSV F induces higher levels of neutralizing IgG than vaccination with post-fusion RSV F (36), while vaccination with pre-fusion-stabilized hMPV F elicited similar neutralizing IgG titers as vaccination with post-fusion hMPV F (40). These data suggest that pre-fusion-specific RSV F antibodies are more prevalent in infected or vaccinated humans and mice, and pre-fusion-specific hMPV F antibodies are present at low levels as compared to antibodies that recognize both pre-fusion and post-fusion hMPV F. The low level of pre-fusion-specific hMPV F antibodies is likely due to a glycan shield near the corresponding RSV site Ø and site V regions on the head of hMPV F.

## Antigenic Epitopes on the RSV F Protein

mAbs binding to the RSV F protein could prevent F protein binding to host cell or hinder the conformational change from pre-fusion to post-fusion, and thus block viral entry into the cell. Due to its sequence conservation, and elicitation of potently neutralizing mAbs, the F protein has become the most popular target for vaccine development. As such, there has been a rapid increase in structural characterization of mAbs in complex with the RSV F protein. Currently available solved structures of mAbs in complex with pneumovirus F proteins are summarized in Table 1. To date, multiple antigenic sites targeted by antibodies have been identified on the RSV F protein (Figure 1). Based on the secondary structure of the protein, six general regions have been designated as antigenic sites: Ø, I, II, III, IV, and V. Among them, antigenic sites I, II, and IV are quite similar between pre-fusion and post-fusion conformations due to their structural conservation upon transition from pre-fusion to post-fusion F. Antigenic sites Ø and V are only present in the pre-fusion conformation (58), while antigenic site III elicits mAbs that are pre-fusion-specific, such as MPE8, while also eliciting mAbs that bind both conformations, such as 25P13 (50). In addition, more than 60% of the most potent neutralizing mAbs bind to sites Ø and V (59), indicating these areas are crucial for immune system recognition and subsequent virus neutralization.

**Table 1:** List of structurally-characterized antibodies in complex with pneumovirus F proteins or fragments thereof.

**mAb**	**PDB ID**	**Origin**	**Antigenic site**	**References**
**RSV F**				
motavizumab	3IXT, 3QWO, 4JLR, 6OE5, 4ZYP	Mouse	II	(44)
101F	3O41, 3O45	Mouse	IV	(45)
hRSV90	5TPN	Human	V	(46)
D25	4JHW	Human	Ø	(36)
MEDI8897	5UDC, 5UDD	Human	Ø	(47)
AM22	6DC5, 6APD	Human	Ø	(48)
5C4	5W23	Mouse	Ø	(49)
MPE8	5U68	Human	III	(50)
ADI19425	6APD	Human	III	(51)
CR9501	6OE4/6OE5	Human	V	(52)
AM14	4ZYP	Human	IV, V	(53)
RSD5	6DC3	Human	Ø	(48)
14N4	5J3D	Human	II	(54)
ADI14359	6APD	Human	I	(51)
R4.C6	6CXC	Mouse	II, IV	(55)
RB1	6OUS	Human	IV	(56)
F-VHH-4	5TOJ	Llama	II, III, IV, V	(57)
F-VHH-L66	5TOK	Llama	II, III, IV, V	(57)
**hMPV F**				
DS7	4DAG	Human	DS7-site	(38)

### Antigenic Site Ø

Antigenic site Ø was the first pre-fusion-specific antigenic site identified on the RSV F protein. The methodology for isolating the first site Ø antibodies was crucial as the mAbs were isolated on the basis of RSV neutralization rather than RSV F protein binding (60). This facilitated the isolation of pre-fusion-specific mAbs without the existence of a pre-fusion RSV F construct. Subsequently, one mAb, D25 (Figure 1C), was utilized to lock the RSV F protein in the pre-fusion conformation (35), which then facilitated stabilization of RSV F in the pre-fusion conformation (36). It is now clear that mAbs that target antigenic site Ø are a large portion of the human B cell repertoire (43, 46, 59). 5C4 is a mAb derived from mice immunized with gene-based vectors encoding the F protein, and is 50 times more potent than palivizumab. Human mAbs D25 and AM22, as well as the mouse mAb 5C4 bind to the apex of the pre-fusion F trimer (site Ø) (36). Importantly, a human mAb based on D25, MEDI-8897, is in clinical trials for prevention of RSV disease in infants (61).

### Antigenic Site V

Antigenic site V was described recently, based on mAb isolation to new pre-fusion-stabilized constructs (46, 59). hRSV90 (Figure 1D) is a site V-targeting human mAb that was found to compete for binding with mAbs that target site II and site Ø. hRSV90 was co-crystallized with the RSV F protein and found to bind just below antigenic site Ø (46). In addition, several site V mAbs were isolated from both adults and infants (51, 59), suggesting these mAbs are prevalent in the human anti-RSV repertoire. CR9501 is a neutralizing mAb isolated from humans, and this mAb was used to demonstrate the dynamic motions of trimeric pre-fusion RSV F protein (52). An antibody that competes for site V of the RSV F protein, MC17, was also shown to cross-react with the hMPV F protein (56).

### Antigenic Site III

The prototypical site III mAb MPE8 (Figure 1E) is unique as it cross-neutralizes multiple viruses in the *Pneumoviridae* family (62). This broad coverage is related to similar V gene usage and somatic mutations in the variable region based on the isolation of a highly similar human antibody 25P13 (50), as well as several other mAbs from a large panel of anti-RSV F human mAbs (59). In addition, site III-specific mAbs are elicited upon initial RSV infection in infants (51). One mAb, ADI19425, which was isolated from an RSV-infected infant, and is potently neutralizing despite lacking substantial somatic hypermutation, was co-crystallized with pre-fusion RSV F (51).

### Antigenic Site II

Palivizumab and motavizumab are the prototypical mAbs to identify antigenic site II on the RSV F protein (26, 44, 63, 64). Targeting antigenic site II of RSV F protein (26), palivizumab is able to neutralize a broad panel of 57 RSV isolates from both subtypes A and B (65). This antigenic site primarily consists of the helix-loop-helix motif of residues 255-275 on the RSV F protein. Several human antibodies have been isolated that bind at antigenic site II (51, 54, 59). The human antibody 14N4 (Figure 1F) was co-crystallized in complex with post-fusion RSV F, and primarily focuses on the 255–275 motif. In the same study, a panel of non-neutralizing mAbs was identified that compete with antigenic site II mAbs on post-fusion RSV F, and suggest some limitations of the palivizumab competition assay used in some vaccine efficacy studies (54). The characterization of mAbs to this antigenic site has led to vaccine candidates focused on antigenic site II (66, 67). Furthermore, serum competition assays with palivizumab have been utilized to characterize vaccine candidates (68). In addition to the mAbs above, nanobodies targeting antigenic site II have been isolated (69, 70), and one nanobody, ALX-0171, has been evaluated as an antiviral therapy to treat RSV infection (71).

### Antigenic Site IV

The site IV epitope is epitomized by the humanized mouse mAb 101F (Figure 1G) (72), and this epitope is structurally conserved between pre-fusion and post-fusion RSV F. The site IV epitope primarily consists of a linear region based on epitope mapping and structural data (45, 73). In addition, it was recently found that 101F cross-reacts with the hMPV F protein (39), presumably by binding to a conserved region at site IV that is similar between RSV and hMPV F (73). Several human mAbs targeting antigenic site IV have also been isolated (51, 59, 73), and human antibody cross-reactivity with hMPV F was correlated to a specific binding pose (73). In addition to the traditional site IV epitope, a mouse mAb, R4.C6, has been isolated that incorporates site IV as well as site II into its epitope (55). The structure of the R4.C6 Fab-post-fusion RSV F complex obtained by cryo-EM showed that the antibody binds to a cross-protomer area in between site II and IV. Recently, a site IV human antibody, RB1, was co-crystallized in complex with pre-fusion RSV F, and a half-life extended variant of this antibody is in clinical development (74).

### Antigenic Site I

The site I epitope on the RSV F protein was identified by the prototypical mouse monoclonal antibody 131-2a (75). Recently, it was determined that human mAbs identified that bind at antigenic site I are weakly or non-neutralizing (51, 54), likely due to insufficient binding to pre-fusion RSV F, as many of these mAbs are post-fusion-specific. The crystal structure of an infant-derived non-neutralizing human mAb, ADI-14359 (Figure 1H), in complex with post-fusion RSV F was determined and defined the antigenic surface for site I (51).

### Other Epitopes and Antibodies

In addition to the epitopes described above, there are several other antibodies isolated that bind unique regions on the RSV F protein. AM14 is a human mAb that recognizes a quaternary epitope spanning two protomers, suggesting the trimeric F protein has specific antigenic epitopes that are not found on the monomeric F protein (53). Single-domain antibody (VHH) or nanobodies from llama immunization were identified and co-crystallized with the RSV F protein (57). Both F-VHH-4 and F-VHH-L66 bind to a cavity in the intermediate area between antigenic site II of one protomer and antigenic site IV of the neighboring protomer. Intranasal administration of these VHHs significantly reduced viral replication in mice, which provides new therapeutic options for antiviral development.

## mAbs Targeting The hMPV F Protein

The first hMPV F-specific neutralizing mAbs generated were derived from immunization of mice and hamsters with various strains of hMPV (76). Of the 12 mAbs in the study, murine mAbs 234 and 338 were effective as passive prophylaxis, protecting mice from hMPV challenge; mAb 338 was successful in reducing lung viral titers when given both prophylactically or therapeutically (77). By generating monoclonal antibody-resistant mutants of antibodies that neutralize hMPV, six antigenic sites of the hMPV F protein were identified (78). Since then, the terminology regarding pneumovirus antigenic sites for hMPV has followed that for RSV. Antigenic sites IV and III from the RSV F protein have been found to be conserved on hMPV F due to the isolation of cross-reactive mAbs discussed in the RSV section. hMPV F-specific mAbs have shown success in neutralizing hMPV both *in vitro* and *in vivo*.

### The DS7-Antigenic Site

A human mAb isolated from a phage display library, termed DS7 (Figure 1I), was shown to reduce hMPV lung viral titers when administered therapeutically in cotton rats (79). mAb DS7 was co-crystallized in complex with a fragment of pre-fusion hMPV F (38), and has a unique molecular footprint in the bottom half of the hMPV F protein. Three additional human mAbs, which are naturally-occurring, termed MPV196, MPV201, and MPV314 were recently isolated and compete for binding with DS7, suggesting these mAbs target the same antigenic site (80).

### Antigenic Site III

The first mAb identified to bind antigenic site III of hMPV F was the cross-reactive human mAb MPE8 (62). As discussed in the RSV section, MPE8 was co-crystallized with the RSV F protein, and the conserved regions at antigenic site III that facilitate cross-reactivity were also hypothesized (50). A similar mAb, 25P13, also discussed above, neutralized hMPV and RSV and competed for binding at antigenic site III (50). Recently, a human mAb, MPV364, was isolated and this mAb competes for binding at antigenic site III, yet does not cross-react with RSV F (80). MPV364 was shown to effectively limit viral replication in BALB/c mice (80). These data suggest antigenic site III can elicit both virus-specific and cross-reactive mAbs. However, the mechanism behind such mAb induction will require additional structural analysis.

### Antigenic Site IV

As discussed earlier, the humanized mouse mAb 101F was identified to cross-react with hMPV F (39). Four human mAbs targeting antigenic site IV of the RSV F protein were isolated, and one mAb, termed 17E10, was identified to also cross-react with hMPV F. This mAb was subjected to peptide mapping and negative-stain microscopy. mAb 17E10 was found to bind a conserved GIIK motif on RSV and hMPV F (39). Furthermore, the binding angle of 17E10 and 101F were shown to be different than non-cross-reactive mAbs, suggesting an altered binding pose is required for cross-reactivity between RSV and hMPV F at antigenic site IV (73).

## Summary and Discussion

In recent years, several breakthroughs have facilitated new knowledge of pneumovirus antibody epitopes. Pre-fusion-stabilized constructs have allowed for isolation of mAbs with optimal neutralization potency, including those binding at antigenic site Ø and V on the RSV F protein. In addition, the use of mAbs to initially lock RSV in the pre-fusion conformation allowed for structure-based design of pre-fusion constructs. While hundreds of mAbs have now been isolated to the RSV F protein, the antigenic epitopes on the hMPV F protein, and related parainfluenza viruses remain unclear. Further studies into antigenic epitopes on these proteins will provide for new insights into pneumovirus immunity and vaccine design. In addition, pre-fusion-stabilized F constructs have now flooded the RSV vaccine field, and there is renewed excitement for the development of an effective RSV vaccine. The field is hopeful that future characterization of mAbs to other pneumovirus surface glycoproteins, as well as assessment of antibody responses to new vaccine candidates will lead to the first safe and effective pneumovirus vaccine.

## Author Contributions

JH, DD, and JM reviewed the literature, and wrote and edited the manuscript.

### Conflict of Interest

The authors declare that the research was conducted in the absence of any commercial or financial relationships that could be construed as a potential conflict of interest.
